# Hypotonicity-induced cell swelling activates TRPA1

**DOI:** 10.1007/s12576-017-0545-9

**Published:** 2017-06-16

**Authors:** Fumitaka Fujita, Kunitoshi Uchida, Yasunori Takayama, Yoshiro Suzuki, Masayuki Takaishi, Makoto Tominaga

**Affiliations:** 1 Division of Cell Signaling, Okazaki Institute for Integrative Bioscience (National Institute for Physiological Sciences), National Institutes of Natural Sciences, Okazaki, Aichi 444-8787 Japan; 2 Basic Research Institute, Mandom Corp., Osaka, 540-8530 Japan; 3 Department of Physiological Sciences, SOKENDAI (The Graduate University for Advanced Studies), Okazaki, Aichi 444-8585 Japan; 4 Laboratory of Advanced Cosmetic Science, Graduate School of Pharmaceutical Sciences, Osaka University, 1-6 Yamadaoka, Suita, Osaka, 565-0871 Japan; 5 Product Assurance Division, Mandom Corp., Osaka, 540-8530 Japan; 6 Institute for Environmental and Gender-Specific Medicine, Juntendo University, Tokyo, 113-0033 Japan

**Keywords:** TRPA1, Hypotonic solution, Cell swelling, Cell-attached patch clamp

## Abstract

Hypotonic solutions can cause painful sensations in nasal and ocular mucosa through molecular mechanisms that are not entirely understood. We clarified the ability of human TRPA1 (hTRPA1) to respond to physical stimulus, and evaluated the response of hTRPA1 to cell swelling under hypotonic conditions. Using a Ca^2+^-imaging method, we found that modulation of AITC-induced hTRPA1 activity occurred under hypotonic conditions. Moreover, cell swelling in hypotonic conditions evoked single-channel activation of hTRPA1 in a cell-attached mode when the patch pipette was attached after cell swelling under hypotonic conditions, but not before swelling. Single-channel currents activated by cell swelling were also inhibited by a known hTRPA1 blocker. Since pre-application of thapsigargin or pretreatment with the calcium chelator BAPTA did not affect the single-channel activation induced by cell swelling, changes in intracellular calcium concentrations are likely not related to hTRPA1 activation induced by physical stimuli.

## Introduction

Hypotonic conditions are known as triggers of various physiological responses, including regulatory volume decreases in human corneal epithelial cells [1] and localized pain in nasal and ocular mucosa [2]. For example, decreases in net transepithelial osmolyte-coupled fluid flux from the cell stroma into tears, which lead to inadequate fluid uptake and subsequent thickening of the stroma, can result in corneal swelling and opacification following injury or infection [3]. In addition, the probability of generalized seizures is increased by water intoxication [4]. Water is also a known irritant in peripheral tissues in tropical immersion foot [5] or aquadynia [6] patients.

Activation of several transient receptor potential (TRP) channels contributes to sensory transduction to produce responses to a wide variety of stimuli, including temperature, nociceptive stimuli, touch, osmolality and pheromones. In particular, the involvement of TRP channels in nociception has been extensively studied following the cloning of the capsaicin receptor TRPV1 [7, 8]. Among thermosensitive TRP channels, TRPV1 [7–9], TRPV2 [10, 11], TRPV4 [12–17], TRPM7 [18, 19] and TRPA1 [20–24] are reported to function as osmo- and mechanosensors that are also activated by temperature and certain chemicals. In a Ca^2+^-imaging experiment, HEK293T cells transiently expressing rat TRPV1 show responses to hypertonic solution [9]. In addition, mouse TRPV2 can detect membrane stretch caused by hypotonic stimulus in mouse aortic myocytes [10]. In rats, pain-related behaviors were induced by application of a hypotonic solution on skin areas that were previously sensitized by PGE_2_, and these pain-related behaviors were reduced following the intrathecal administration of *Trpv4* antisense RNA [12], although the role of TRPV4 in nociception is still a matter of debate. On the other hand, TRPM8 [25, 26] is activated by increases in osmolality over physiological levels and inhibited by reduced osmolality in mice, suggesting that TRPM8 may be involved in regulating eye blinking in response to various peripheral osmolalities [27].

TRPA1 is thought to be involved in detecting a variety of chemical stimuli, including allyl isothiocyanate (AITC), a main component of mustard oil [20, 28–31]. We also previously identified TRPA1 as a receptor for skin irritants such as parabens [32] and alcohols [33], as well as for pain-producing alkaline pH [34]. In addition, TRPA1 agonists induce secretion of disease-associated mucins in human nasal passages in vivo [35]. Thus, TRPA1 is accepted as an ion channel that is related to acute nociception and inflammatory pain, and is considered to be a promising target for the development of analgesic agents. Indeed, many human TRPA1 (hTRPA1) antagonists have been developed or isolated from natural compounds, including HC-030031, CMP1 and 1,8-cineole, as well as camphor and its analogues [36–41]. On the other hand, a role for TRPA1 in sensing noxious cold stimuli and somatic mechanosensation in vivo remains unsettled, especially in mammals [21, 30–38]. A recent report shows the possibility that human and mouse TRPA1 contribute to sensing warmth and uncomfortable heat in addition to noxious cold [42]. TRPA1 was reported to play a role in mechanical hyperalgesia [20], and hyperosmotic stimulation directly activated rat TRPA1 in both transfected cells and native sensory neurons at a single-channel level [22]. In addition, mouse TRPA1 reportedly modulates mechanotransduction via a cell-autonomous mechanism in nociceptor termini [21]. Recent studies involving human and rat odontoblasts showed that a known TRPA1 blocker inhibited currents induced by hypotonic solutions, raising the possibility that hTRPA1 can respond to hypotonic solutions [23, 24]. However, the role of this channel in mechanosensation and in sensing hypotonic conditions is not well defined.

We found that hypotonic solutions induced and enhanced hTRPA1 activation in an intracellular calcium-independent manner when heterologously expressed in human embryonic kidney-derived 293T (HEK293T) cells. Moreover, we determined the correlation between increases in cell volume and TRPA1 activation under hypotonic conditions using a cell-attached patch-clamp technique.

## Materials and methods

### Cell cultures

HEK293T cells were maintained in DMEM (WAKO Pure Chemical Industries, Ltd., Osaka, Japan) supplemented with 10% FBS (Biowest SAS, Caille, France), 100 units/ml penicillin (Thermo Fisher Scientific Inc., Carlsbad, CA, USA), 100 µg/ml streptomycin (Thermo Fisher Scientific Inc.) and 2 mM l-glutamine (GlutaMAX, Thermo Fisher Scientific Inc.) at 37 °C in 5% CO_2_. For Ca^2+^-imaging, 1 µg pcDNA3.1 plasmid DNA carrying human *TRPA1* (obtained from Life Technologies, Carlsbad, CA, USA) in OPTI-MEM medium (Thermo Fisher Scientific Inc.) was transfected into HEK293T cells using Lipofectamine Plus Reagent (Thermo Fisher Scientific Inc.). After incubating for 3–4 h, the cells were reseeded on cover slips and further incubated at 37 °C in 5% CO_2_.

### Ca^2+−^ imaging

HEK293T cells on cover slips were mounted in an open chamber and superfused with standard bath solution (140 mM NaCl, 5 mM KCl, 2 mM MgCl_2_, 2 mM CaCl_2_, 10 mM HEPES, 10 mM glucose, pH 7.4). Several minutes before the experiments, the standard bath solution was changed to ±0 mOsm isotonic solution (60 mM NaCl, 5 mM KCl, 2 mM MgCl_2_, 2 mM CaCl_2_, 10 mM HEPES, 160 mM mannitol, 10 mM glucose, pH 7.4). Hypotonic solutions at various tonicities were adjusted by mannitol with −160 mOsm hypotonic solution (60 mM NaCl, 5 mM KCl, 2 mM MgCl_2_, 2 mM CaCl_2_, 10 mM HEPES and 10 mM glucose, pH 7.4). To make a Ca^2+^-free bath solution, CaCl_2_ was omitted and 5 mM EGTA was added. Osmolalities of the calcium-containing solutions defined as 0, −80, −120 and −160 mOsm were directly measured with a osmometer (OM-815, Vogel Medizintechnik, Germany), which yielded 300, 216, 176 and 135 mOsm, respectively, whereas the osmolalities of the calcium-free solution defined as 0 and −120 mOsm were measured as 306 and 184 mOsm, respectively. Because the calculated osmolalities were close to the measured values, we used the calculated values. Cytosolic free Ca^2+^ concentration ([Ca^2+^]_i_) in HEK293T cells was measured by dual-wavelength fura-2 (Thermo Fisher Scientific Inc.) microfluorometry with excitation at 340/380 nm and emission at 510 nm. In time-lapse measurements, the cells were maintained at each osmolality for 100 s with or without 1 μM AITC. The ratio image was calculated and acquired using the IP-Lab imaging processing system (Scanalytics Inc., Fairfax, VA USA). To chelate intracellular Ca^2+^, cells were treated with 10 μM BAPTA-AM (Dojindo, Kumamoto, Japan) for 1 h before Ca^2+^-imaging measurements.

### Electrophysiology

Cell-attached patch-clamp recordings were performed 1 day after transfection. The standard bath solution was the same as that used in the Ca^2+^-imaging experiments. The pipette solution contained 140 mM KCl, 5 mM EGTA and 10 mM HEPES, pH 7.4 (adjusted with KOH), as previously reported [10]. Data from cell-attached voltage-clamp recordings were sampled at 10 kHz and filtered at 5 kHz with a low-pass filter for analysis (Axon 200B amplifier with pCLAMP software). The pipette potential was held at −60 mV. All experiments were performed at room temperature. NPo values were calculated from representative current traces 20–30 s after each stimulus that lasted for 10 s by pCLAMP software (Axon Instruments, Sunnyvale, CA, USA). The total number of channels and total events in each analysis ranged from 1 to 7 and from 2 to 6653, respectively.

### Cell volume measurement

Cell volume was estimated by measuring the maximal cross-sectional area of cells (cA) captured by digital camera (P6000; Nikon, Tokyo, Japan) in digitized images using Image J (U.S. National Institutes of Health, Bethesda, MD, USA) and a pen tablet (PTH-450; Wacom, Saitama, Japan) to define the cross-sectional area. All cA values were measured during the control period at neutral tonicity and the subject period at various hypotonic conditions. Area increase ratios were estimated by the following equation: ${\text{Ratio = cA}}_{\text{hypotonic}} / {\text{cA}}_{\text{control}}$.

### Data analysis

Data are expressed as mean ± SEM. Statistical analyses were performed by Student’s *t*-test or one-way analysis of variance (ANOVA) followed by a two-tailed multiple *t*-test with Bonferroni correction. *p* values less than 0.05 were considered significant.

## Results

### Enhancement of TRPA1 activity under hypotonic conditions

**Fig. 1:**
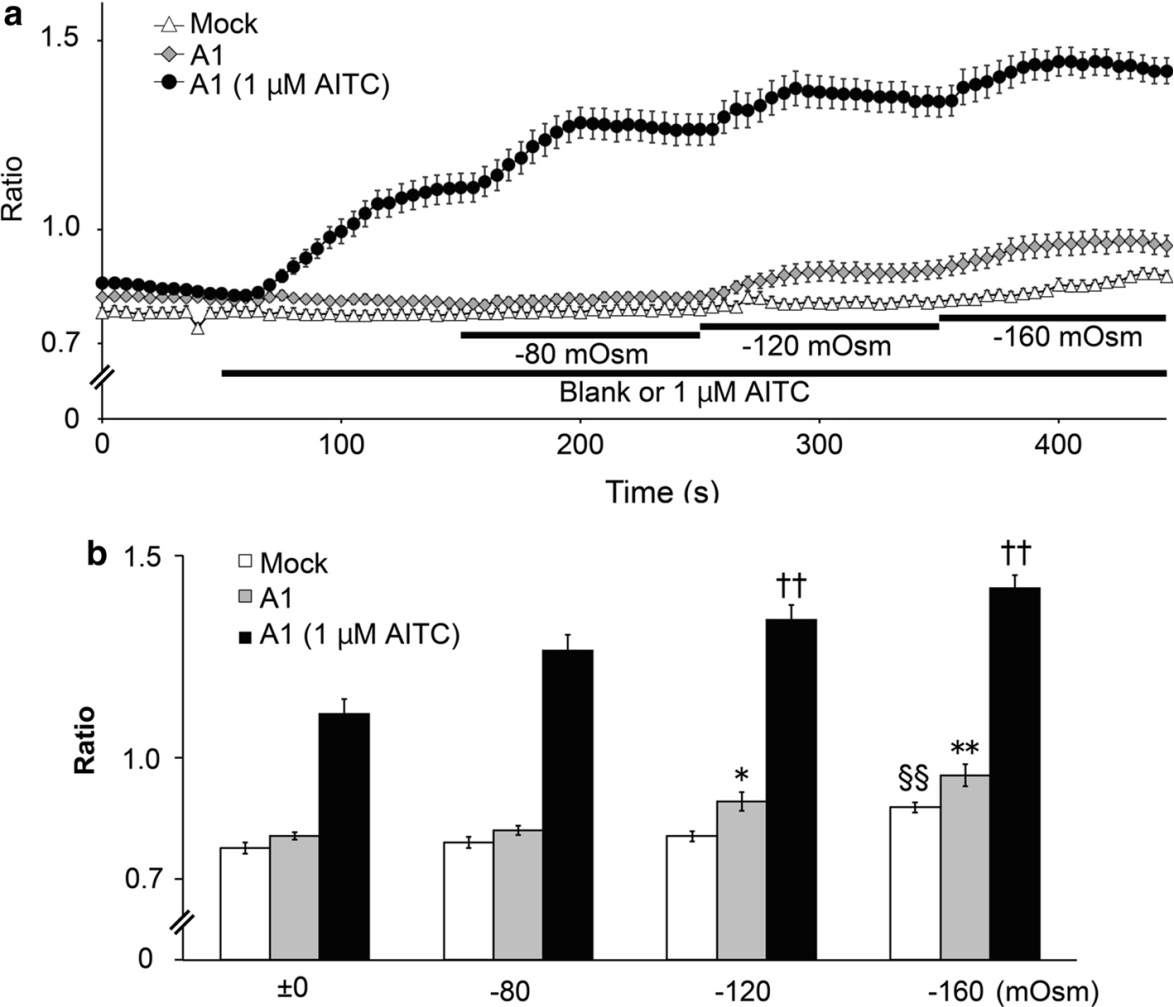
Hypotonic solutions induced an increase in cytosolic calcium concentrations in cells expressing human TRPA1. **a** Changes in cytosolic Ca^2+^ concentrations with time. Data are presented as the fura-2 ratio (340/380 nm) in HEK293T cells expressing human TRPA1(A1) or cells transfected with pcDNA3.1 (Mock) (*N* = 72) in the presence of extracellular Ca^2+^ after 1 min exposure to hypotonic solutions with (*N* = 93) or without (*N* = 93) 1 μM AITC. **b** Hypotonic solutions increased cytosolic Ca^2+^ concentrations. Comparison of the fura-2 ratio changes at the end of each solution application. ^§§^ *p* < 0.01 vs ±0 mOsm in the mock-transfected cells. *p < 0.05; **p < 0.01 vs 0 mOsm in *hTRPA1*-transfected cells without 1 μM AITC. ^††^ *p* < 0.01 vs 0 mOsm in *hTRPA1*-transfected cells with 1 μM AITC

We first used a Ca^2+^-imaging method to examine whether hypotonic solutions activate TRPA1 in HEK293T cells expressing hTRPA1 (Fig. 1a, b). Treatment of cells with hypotonic solutions at −120 and −160 mOsm below the normal isotonic osmolality significantly increased [Ca^2+^]_i_ in cells expressing hTRPA1 (Fig. 1a, b). In addition, increases in [Ca^2+^]_i_ induced by application of 1 μM allyl isothiocyanate (AITC) were significantly enhanced at −120 and −160 mOsm in hTRPA1-expressing cells. These results suggested that hTRPA1 activity was increased under hypotonic conditions. Because a significant increase in [Ca^2+^]_i_ was detected under −160 mOsm hypotonic conditions, even in the mock-transfected cells, we used −120 mOsm hypotonic conditions for most of the subsequent experiments.

**Fig. 2:**
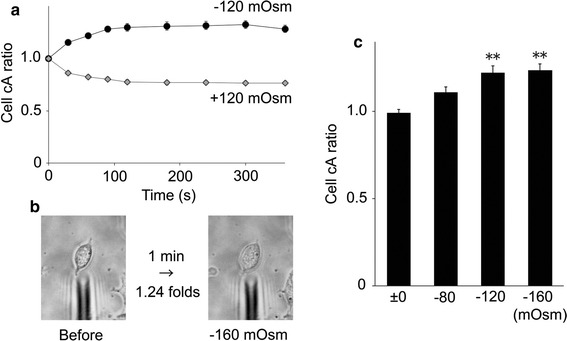
Cross-sectional area (cA) changes in HEK293T cells induced by hypotonic stimuli. **a** Average cell cA changes by −120 mOsm (*N* = 10) hypotonic and +120 mOsm (*N* = 10) hypertonic stimuli. **b** Representative images of cell swelling by −160 mOsm hypotonic stimulation for 1 min. **c** Comparison of cA increase ratio for cells exposed to hypotonic solutions with different osmolalities (±0 mOsm, *N* = 5; −80 mOsm, *N* = 7; −120 mOsm, *N* = 5; −160 mOsm, *N* = 8) **p < 0.01

**Fig. 3:**
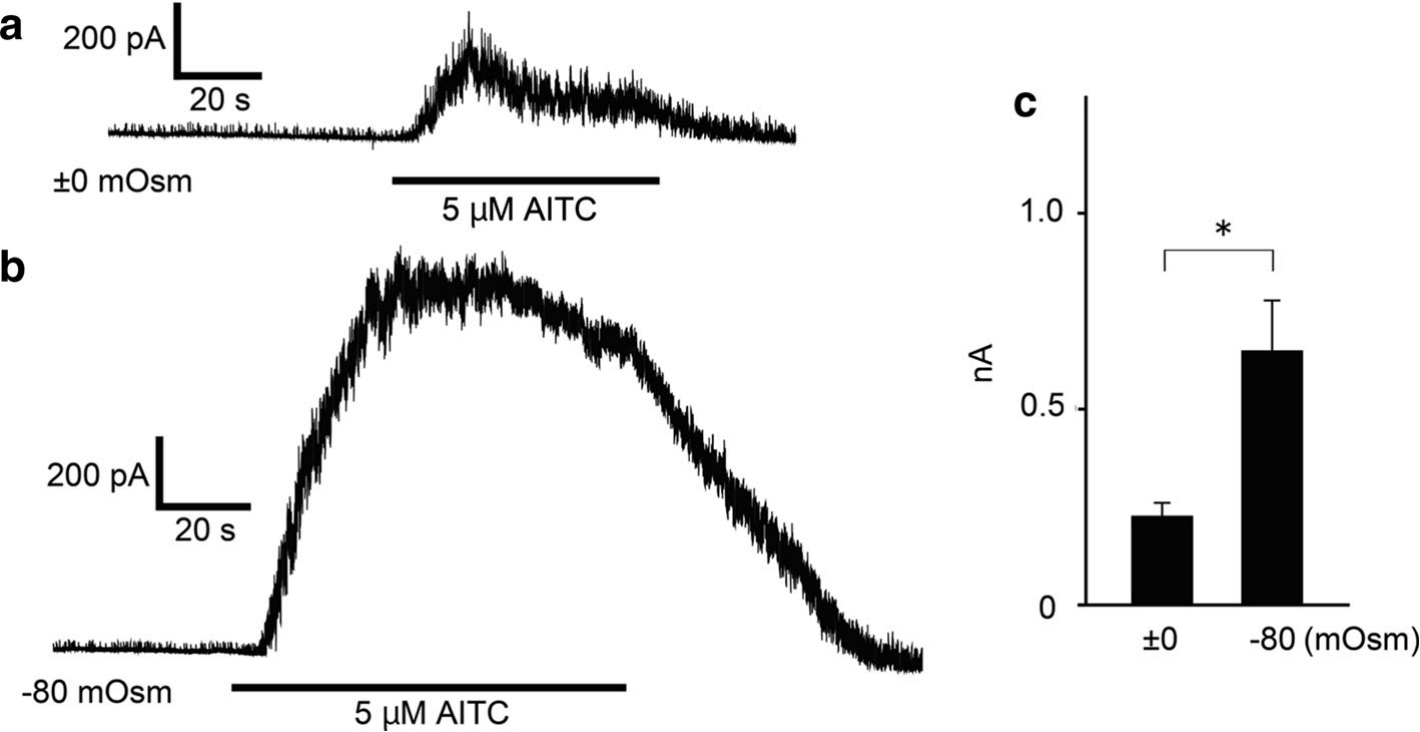
Hypotonic solutions increased AITC-evoked currents. **a** A representative trace of hTRPA1-mediated single-channel currents activated by 5 μM AITC in ±0 mOsm isotonic bath solution in a cell expressing hTRPA1. **b** A representative trace of hTRPA1-mediated single-channel currents activated by 5 μM AITC in −80 mOsm hypotonic solution in a cell expressing hTRPA1. **c** Comparison of hTRPA1 currents activated by 5 μM AITC under ±0 mOsm isotonic conditions (*N* = 6) and −80 mOsm hypotonic conditions (*N* = 6). ^*^ *p* < 0.05

Since hypotonic conditions induce cell swelling to produce an increase in membrane tension that could be a mechanical stimulus for proteins localized in the extended plasma membrane, we examined the hTRPA1 activity in HEK293T cells with a patch-clamp method. Upon hypotonic stimulation of HEK293T cells, swelling lasted for several minutes before the onset of a regulatory volume decrease (Fig. 2) [43, 44]. Therefore, we completed all patch-clamp recordings within 5 min. We chose a cell-attached configuration to preserve an intact cytosolic environment. After making a cell-attached configuration, we applied a hypotonic solution of −80 mOsm, which alone did not cause [Ca^2+^]_i_ increases in the cells (Fig. 1a, b). As expected, the basal channel activities were similar under both ±0 and −80 mOsm conditions. However, the increases in channel activity following 5 μM AITC application were significantly larger under −80 mOsm conditions relative to isotonic conditions (Fig. 3a–c). It is noted that hTRPA1-mediated single-channel currents were observed even before application of AITC, as previously reported [34]. These data indicated that the hypotonic conditions enhance hTRPA1 channel activity.

### Activation of TRPA1 by cell swelling under hypotonic conditions

**Fig. 4:**
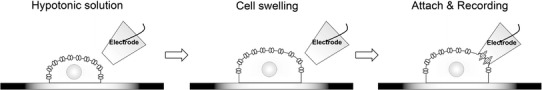
Schematic diagram of “a post-attached method”. A patch pipette was attached to the plasma membrane after cell swelling induced by exposure to hypotonic solutions

**Fig. 5:**
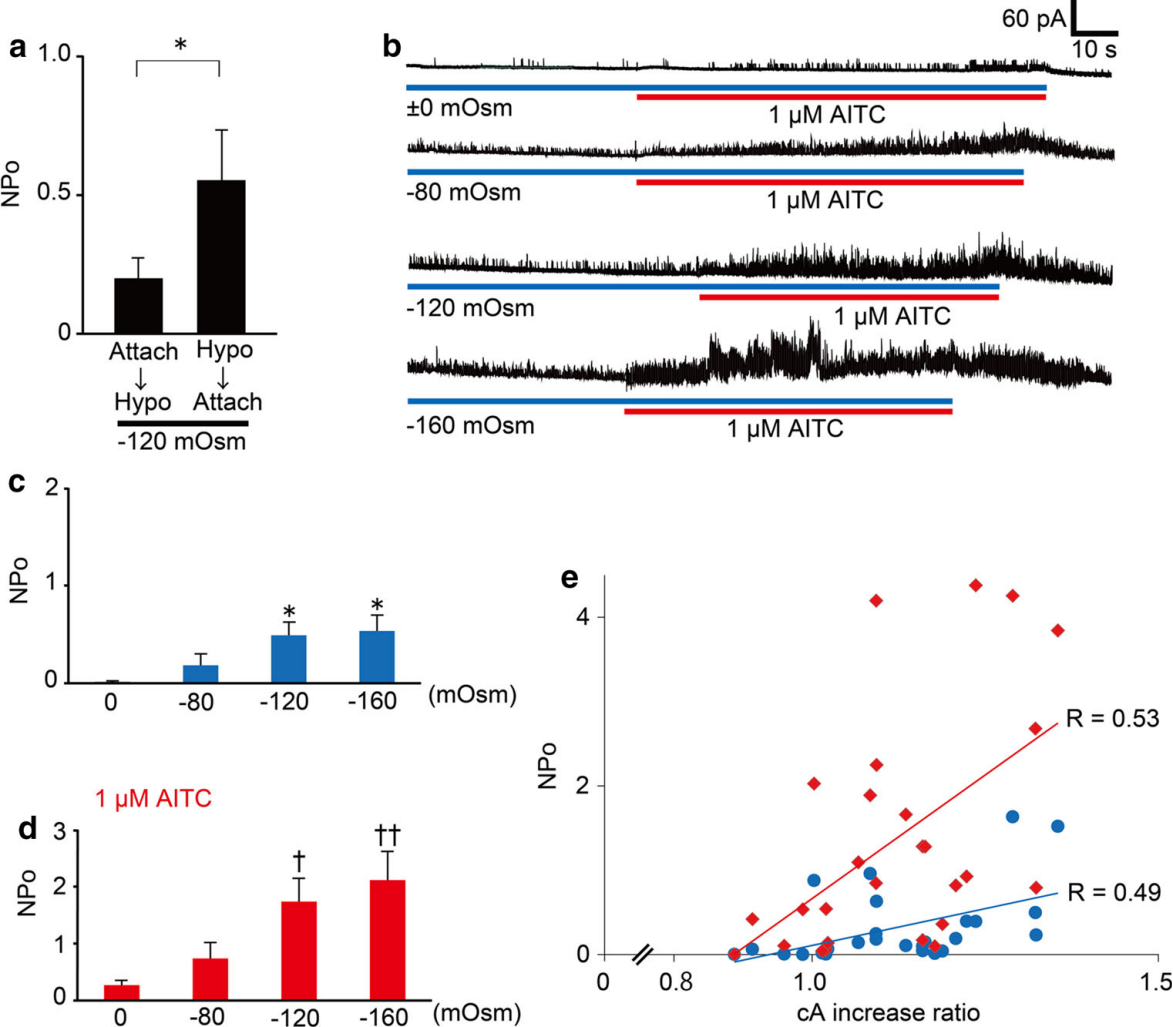
Cell swelling induced by hypotonic solution activated hTRPA1. **a** Comparison of NPo values for single-channel currents activated by hypotonic stimulus in cells expressing hTRPA1 upon establishing a cell-attached configuration before (*N* = 7) and after (*N* = 11) exposure to −120 mOsm hypotonic solutions. *p < 0.05. **b** Representative traces of the single-channel currents activated by hypotonic stimulus in cells expressing hTRPA1 without and with AITC. **c** NPo values of single-channel currents in cells expressing hTRPA1 that were exposed to hypotonic solutions of different osmolalities (±0 mOsm, *N* = 8; −80 mOsm, *N* = 7; −120 mOsm, *N* = 11; −160 mOsm, *N* = 9). *p < 0.05 vs ±0 mOsm. **d** NPo values of single-channel currents in cells expressing hTRPA1 that were exposed to hypotonic solutions of different osmolalities (±0 mOsm, *N* = 8; −80 mOsm, *N* = 7; −120 mOsm, *N* = 10; −160 mOsm, *N* = 8) with 1 μM AITC. ^†^ *p* < 0.05; ^††^ *p* < 0.01 vs ±0 mOsm. **e** Correlations between cA increase ratios and NPo values in cells expressing hTRPA1 that were exposed to hypotonic solutions (*black circles* without AITC, *N* = 24; *black diamonds* with AITC, *N* = 24)

In theory, during cell-attached patch-clamp experiments, the tension of the membrane patch should remain constant as hypotonic solutions are applied to the patched cell. To examine the membrane tension in the patch, we first compared the single-channel activities using two methods. In one method, cells were exposed to the −120 mOsm hypotonic solution after making a cell-attached configuration (a pre-attached method). In the other method, a patch pipette was attached to the plasma membrane 1 min after exposure to the −120 mOsm hypotonic solution (a post-attached method, Fig. 4) when HEK293T cells were swollen (Fig. 2a). NPo (open probability x open channel number) values of the hTRPA1-mediated currents were significantly larger in the post-attached method than in the pre-attached method (Fig. 5a), suggesting that increases in membrane tension activated hTRPA1. In addition, we did not observe any single-channel current activation with the post-attached method in mock-transfected cells exposed to the −120 mOsm hypotonic solution (*N* = 3, data not shown). Accordingly, we used the post-attached method in subsequent experiments. In these experiments, isotonic solution (±0 mOsm) did not induce cell swelling (average cA increase ratio was 0.99) while −80, −120 and −160 mOsm hypotonic solutions induced cell swelling in a hypotonicity-dependent manner (average cA increase ratios were 1.11, 1.22 and 1.24, respectively, Fig. 2b, c). Single-channel currents were increased by hypotonic stimulus in a hypotonicity-dependent manner with statistical significance at osmolalities ≤ −120 mOsm (Fig. 5b, c); this pattern was similar to that seen for changes in cell cA (Fig. 2b, c). In addition, the single-channel currents upon application of 1 μM AITC also became larger in a hypotonicity-dependent manner, achieving statistical significance at osmolalities ≤ −120 mOsm (Fig. 5b, d). These results suggested that hTRPA1 activity is not only enhanced but also induced by the hypotonic conditions.

**Fig. 6:**
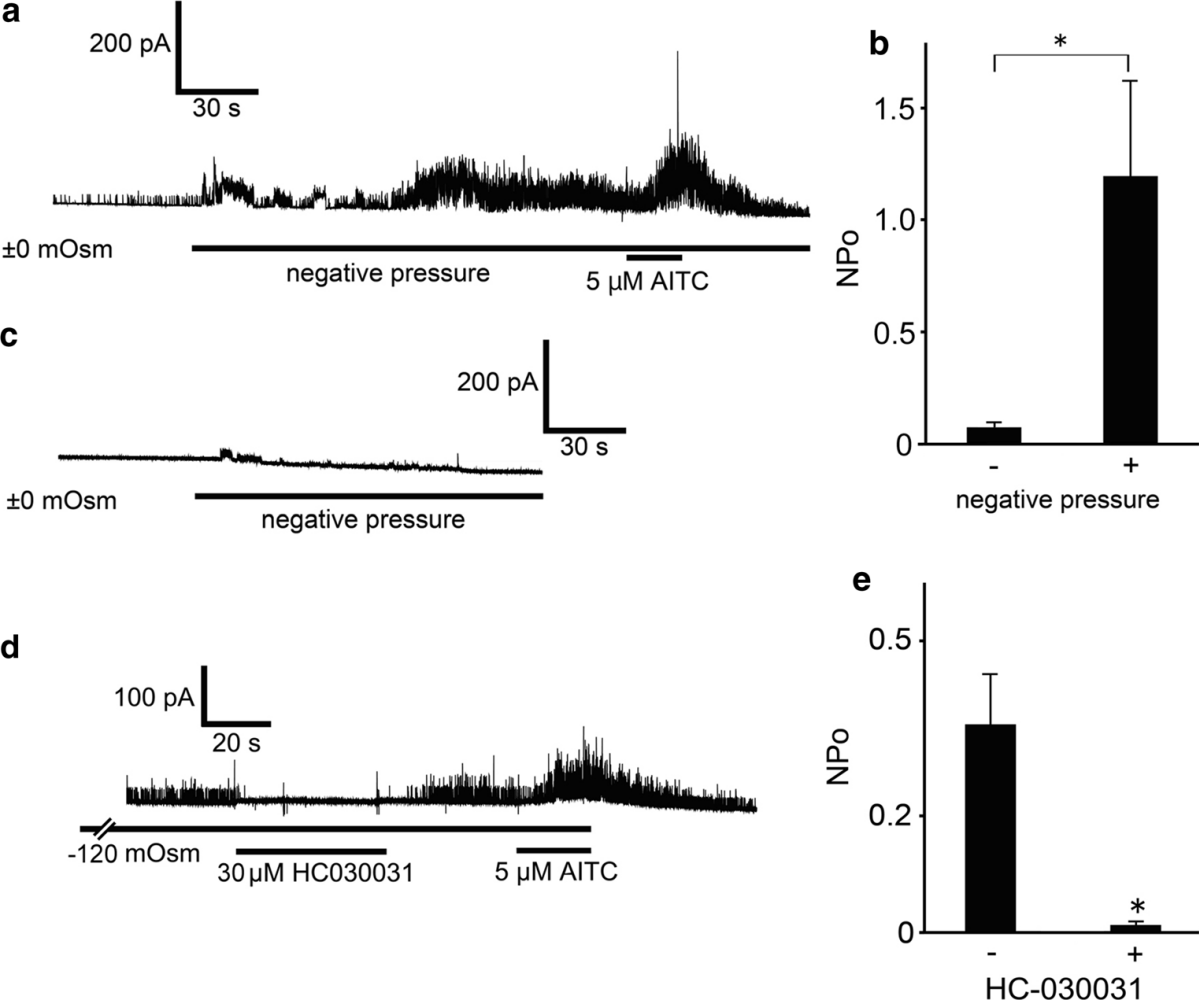
hTRPA1-mediated single-channel currents activated by negative pressure to the pipette or a hypotonic solution. **a** A representative trace of hTRPA1-mediated single-channel currents activated by negative pressure, which was adjusted to approximately 6.6 cmHg with a microliter #702 syringe (HAMILTON Company Inc., Nevada, USA). **b** Comparison of hTRPA1 currents with or without negative pressure. *p < 0.05. **c** Representative trace of the currents activated by negative pressure applied to a mock-transfected cell. **d** A representative trace of hTRPA1-mediated single-channel currents in the presence of the TRPA1 inhibitor HC-030031 (30 μM). **e** Inhibitory effects of HC-030031 (*N* = 6) on hTRPA1-mediated single-channel currents induced by hypotonic solution. *p < 0.05

To better estimate the influence of cell swelling on hTRPA1 activities under hypotonic conditions, we analyzed the correlation between increases in cell cA and hTRPA1 activities. Positive correlations between cA increase ratios and NPo values of hTRPA1-mediated single-channel currents were observed with (*R* = 0.53) or without (*R* = 0.49) 1 μM AITC (Fig. 5e), suggesting that cell swelling under hypotonic conditions induced hTRPA1 activation. Relatively modest hypotonic stimulation is known to result in unfolding of membrane invaginations without producing mechano-stress [45–47]. To confirm whether mechanical stress on the patch membrane evoked by negative pressure causes hTRPA1-mediated current responses, we performed cell-attached patch-clamp experiments with negative pressure applied to the patch pipette in cells expressing hTRPA1. Approximately 6.6 cmHg negative pressure significantly increased NPo values of hTRPA1 currents under isotonic conditions, whereas small TRPA1 channel currents were observed before application of negative pressure (Fig. 6a, b). Since obvious TRPA1 currents were not observed under negative pressure in mock-transfected cells (Fig. 6c), the increased currents under negative pressure were likely derived from hTRPA1 activation. These data support our hypothesis that hTRPA1 is activated by mechano-stress.

### Effects of hTRPA1 blockers and cytosolic calcium on hypotonicity-activated single-channel hTRPA1 currents

Next we examined the effects of known general TRPA1 blockers [48] on hypotonicity-activated single-channel currents to confirm that the observed channel activity is attributable to TRPA1 expression in HEK293T cells. The selective TRPA1 antagonist HC-030031 (30 μM) caused almost complete inhibition of hypotonic solution (−120 mOsm)-induced single-channel currents, and the inhibition was reversed by washout (Fig. 6d, e). Meanwhile, the TRPA1 activator AITC (5 μM) increased the channel activity similar to that shown in Fig. 5. However, ruthenium red, a hydrophilic TRPA1 blocker, showed little inhibition (*N* = 5, data not shown). These different inhibitory effects of TRPA1 blockers suggested that blockers must penetrate the plasma membrane and reach the cell-attached patch membrane from the inside.

**Fig. 7:**
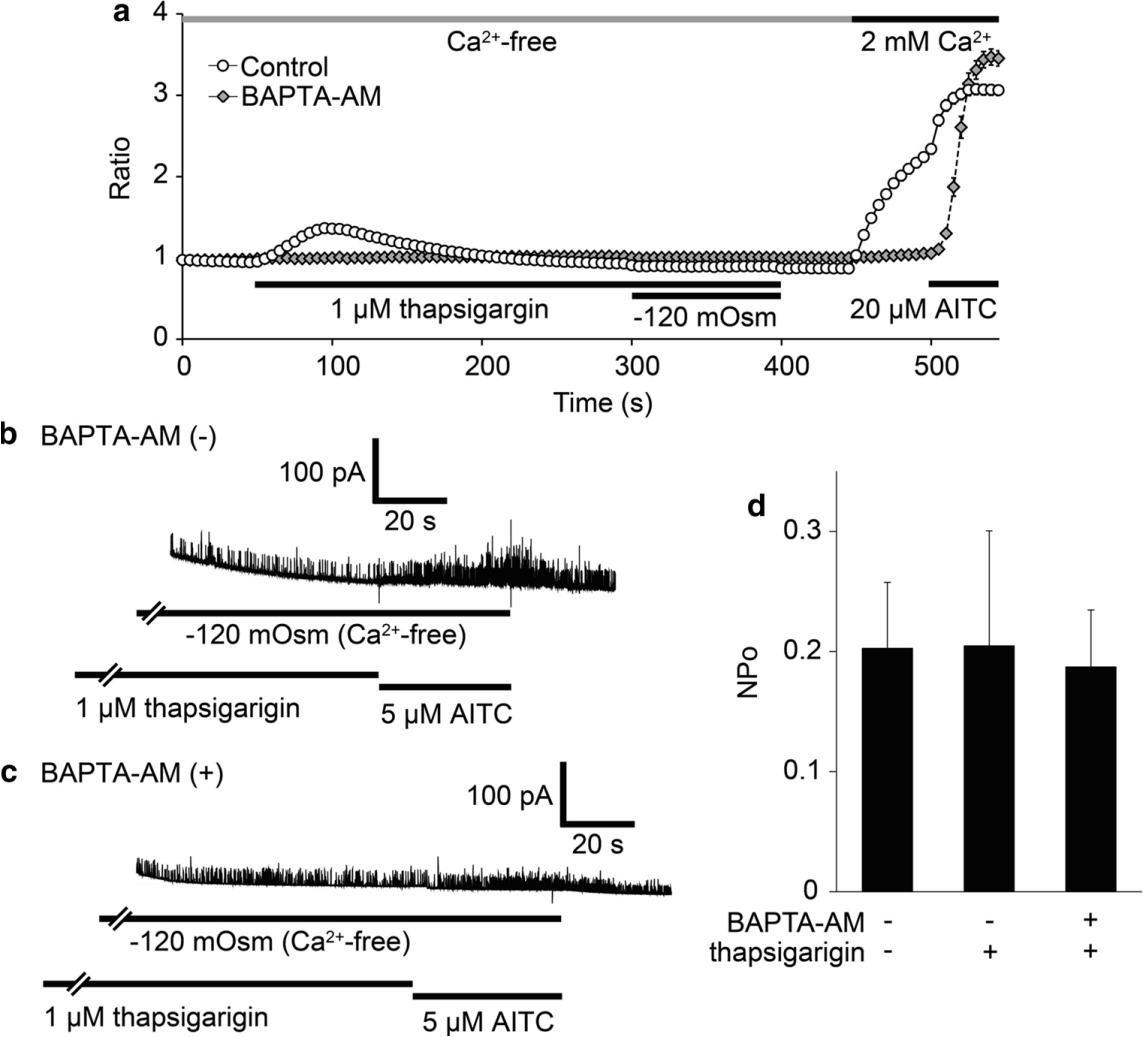
Effects of cytosolic calcium on single-channel currents activated by a hypotonic solution. **a** Average traces of fura-2 ratio changes in hTRPA1-expressing cells pre-loaded with BAPTA-AM or treated with thapsigargin, and the effects of hypotonic stimulus (−120 mOsm) in the absence of extracellular Ca^2+^. Data are from cells with (*N* = 47) or without (*N* = 116) 10 μM BAPTA-AM. **b, c** Representative traces of hTRPA1-mediated single-channel currents in HEK293T cells exposed to 1 μM thapsigargin (3 min) before hypotonic stimulus in the absence of extracellular Ca^2+^ without (**b**) or with (**c**) preloading with 10 μM BAPTA-AM. **d** Comparison of hTRPA1-mediated single-channel currents activated by hypotonic solutions with (*N* = 11) or without (*N* = 17) pre-exposure to 1 μM thapsigargin and single-channel currents in BAPTA-AM pre-loaded cells (*N* = 11)

TRPA1 is activated directly by intracellular calcium via binding to its putative EF-hand-like motif in the N-terminal region [49, 50]. [Ca^2+^]_i_ was previously shown to be increased under hypotonic conditions even in HEK293T cells without heterologous expression of any other channel [51, 52]. We observed similar increases in [Ca^2+^]_i_ although it was smaller relative to those seen for cells expressing hTRPA1 (Fig. 1a, b). To clarify whether hTRPA1 activation under hypotonic conditions was induced by increases in intracellular Ca^2+^ levels, we examined the effect of thapsigargin, which is known to deplete intracellular Ca^2+^ levels by inhibiting SERCA, in the absence of extracellular Ca^2+^ with or without 10 μM BAPTA-AM treatment for 1 h. Small and transient [Ca^2+^]_i_ increases were induced by the application of 1 μM thapsigargin in the absence of extracellular Ca^2+^, which could be due to the block of Ca^2+^ uptake to the ER by SERCA (Fig. 7a). On the other hand, in cells treated with BAPTA-AM hardly any change in [Ca^2+^]_i_ was observed upon thapsigargin application (Fig. 7a). In cells pre-exposed to thapsigargin (1 μM) for 3 min, hTRPA1-mediated single-channel currents showed NPo values that were similar to the control (Fig. 7b, d). BAPTA-AM treatment also did not affect the current activation under −120 mOsm hypotonic conditions (Fig. 7c, d). These results indicated that changes in cytosolic Ca^2+^ concentrations are likely not involved in the observed hTRPA1-mediated current activation following exposure of cells to hypotonic conditions.

## Discussion

In the present study, Ca^2+^-imaging and cell-attached patch-clamp experiments clearly showed that hypotonic stimulation induced TRPA1 activation and enhanced currents activated by AITC. In addition, cell swelling promoted by hypotonic conditions activated TRPA1 in a manner that was independent of cytosolic calcium.

During the Vietnam War, the potential of water to be an irritant in peripheral tissues was seen with so-called tropical immersion foot that affected soldiers whose feet were immersed in water for prolonged periods [5]. Water also causes pruritus, paresthesia and pain in patients diagnosed with aquadynia [6], although the molecular mechanisms that cause these pain sensations remain unknown. Our findings suggest the possibility that, in addition to its previously defined roles in mechano-sensation in various organs, TRPA1 participates in nociception upon exposure to hypotonic solutions such as water.

Our findings in this study suggest that hTRPA1 activity was increased under hypotonic conditions, a result that was inconsistent with previous data in which hypertonic, and not hypotonic, stimuli caused TRPA1 activation in rat cells [22]. This inconsistency might be partly due to species differences. Indeed, human and rat TRPA1 exhibited opposite responses to menthol [39] and protons [36]. Moreover, two studies [40, 42] that used rat and human odontoblasts exposed to hypotonic solutions showed a possible role for TRPA1 in detecting hypotonic conditions in that the responses could be inhibited by known TRPA1 agonists. These findings could support our present results and indicate that TRPA1 is indeed involved in hypotonic responses. The post-attached method we utilized in which a gigaohm seal for the cell-attached patch is introduced to the cell membrane after exposure to the hypotonic solution might also have caused different outcomes. If our method more precisely reflects actual membrane conditions, this approach could be helpful for future studies of mechano-sensitive ion channels, including TRP channels.

In the cell-attached patch-clamp experiments, hTRPA1 single-channel currents were enhanced by 5 μM AITC (Fig. 3a–c); however, data from Ca^2+^-imaging with AITC showed small differences between isotonic and hypotonic conditions (Fig. 1a, b). Although small intracellular Ca^2+^ increases were indeed induced by hypotonic stimulation, such small intracellular Ca^2+^ increases beneath the patch-clamped membrane could be sufficient to cause large synergistic activation of hTRPA1 with AITC. In addition, there was slightly more tension in the cell-attached patch-clamped membrane, which was required to achieve a gigaohm seal. This tension might also promote TRPA1 activation.

TRPA1 expression in mouse inner ears and electro-physiological data seemed to supply strong evidence that TRPA1 has an obvious role as a mechano-receptor in mechano-transduction [53]. On the other hand, results from studies of TRPA1 knockout mice were inconsistent with in vitro findings [54], while cold responses of mouse TRPA1 have become more convincing [21, 37]. Therefore, the roles of TRPA1 as a mechano-receptor still remain to be clarified both in vitro and in vivo. However, our result demonstrating single-channel hTRPA1 activation upon hypotonic stimulus strongly supports a potential role for TRPA1 as a mechano-receptor in vitro. The similarity between the time course of cell cA changes (Fig. 2a) and [Ca^2+^]_i_ changes (Fig. 1a) supports the notion that TRPA1 activation occurs immediately following cell volume changes that likely create membrane stretch.

In addition to TRPA1, several other TRP channels, including TRPC1 [55], TRPM2 [56], TRPM7 [18, 19], TRPV2 [10, 11] and TRPV4 [57], are reported to be activated by mechanical stimulus, although whether these TRP channels are activated directly by mechanical stimulus or via other intracellular components is not clearly understood. Nevertheless, our finding that human TRPA1 shows activation at a single-channel level with the post-attached method but not in the pre-attached method could rule out the involvement of intracellular components. Further study will be required to confirm whether hypotonicity-dependent hTRPA1 activation occurs independently of other factors.
